# The Spin of Electrons and the Proof for the Action of Homeopathic Remedies

**DOI:** 10.25122/jml-2020-0140

**Published:** 2020

**Authors:** George Vithoulkas, Camelia Berghian-Grosan

**Affiliations:** 1. University of the Aegean, Mytilene, Greece; 2. National Institute for Research and Development of Isotopic and Molecular Technologies, Cluj-Napoca, Romania

**Keywords:** Electron spin, vital force, electromagnetic influences, levels of health, homeopathy

## Abstract

In the last 200 years, the action of the highly diluted homeopathic remedies has been proved by their curative effect on the human organism. In this work, a hypothesis concerning the mystifying question about this action is proposed. The hypothesis suggests that any pathology, either functional or structural, can be detected in the change of the overall energy of the human body. Such energy is constituted by fields of force according to quantum physics. More precisely, every disturbance of the human organism affects the spin on electrons of different elements within the human body, and their reset could take place with an agent similar to the electromagnetic force that created the problem. This statement has been proved by the correct homeopathic treatments, as it can be seen in many published cases. The hypothesis is based on two approaches, the idea of the spin of electrons and the vital force, and their scientific relevance.

## Introduction

### The idea of the spin of electrons

It is well-known that electrons go around the nucleus but, also, they spin around their axis in accord with their intrinsic angular momentum [1]. 

Coupled electron spins are stable, but can be changed under electromagnetic influences [2, 3]. The logical conclusion, therefore, is that if the spin momentum changes, there must be a change in the properties or behavior of the relevant atom within the human body.

### The vital force

The matter is a combination of elements; the smallest particle of the elements with distinctive chemical properties is the atom. Living organisms are built of only a few common elements: carbon (C), hydrogen (H), nitrogen (N), and oxygen (O) and less common elements: sodium (Na), magnesium (Mg), phosphorus (P), sulfur (S), chlorine (Cl), potassium (K), calcium (Ca) of the known elements [4]. To these, some metals ions, known as “trace elements”, like cobalt, copper, iron, manganese, molybdenum and zinc, are also indispensable for human beings’ life [5]. The next levels in the hierarchy are the molecules and then, as a combination of molecules, the materials or larger structures [4, 6]. The occurrence of these combinations depends on the information carried by atoms and molecules. The information is related to the arrangements of electrons around the nucleus and, implicitly, to the types of bonds that are possible. On the other part, the molecules possess information about the possible arrangements of atoms to form it [6]. Thus, the outermost electrons determine how the atoms interact; the atoms are carrying this information and influence the molecules and, finally, the characteristics of the cell [4]. They influence each other on a subatomic level and, actually, they form an overall field of force that in homeopathy is called vital force [7] or magnetic forces in other medical fields [8]; the idea of vitalism persisting over the years in the biological fields and beyond [9]. The vital force contains all the information concerning the mental, emotional and physical levels. The vital force receives information from the external and internal environment and responds to such stimuli [10].

**Figure 1:**
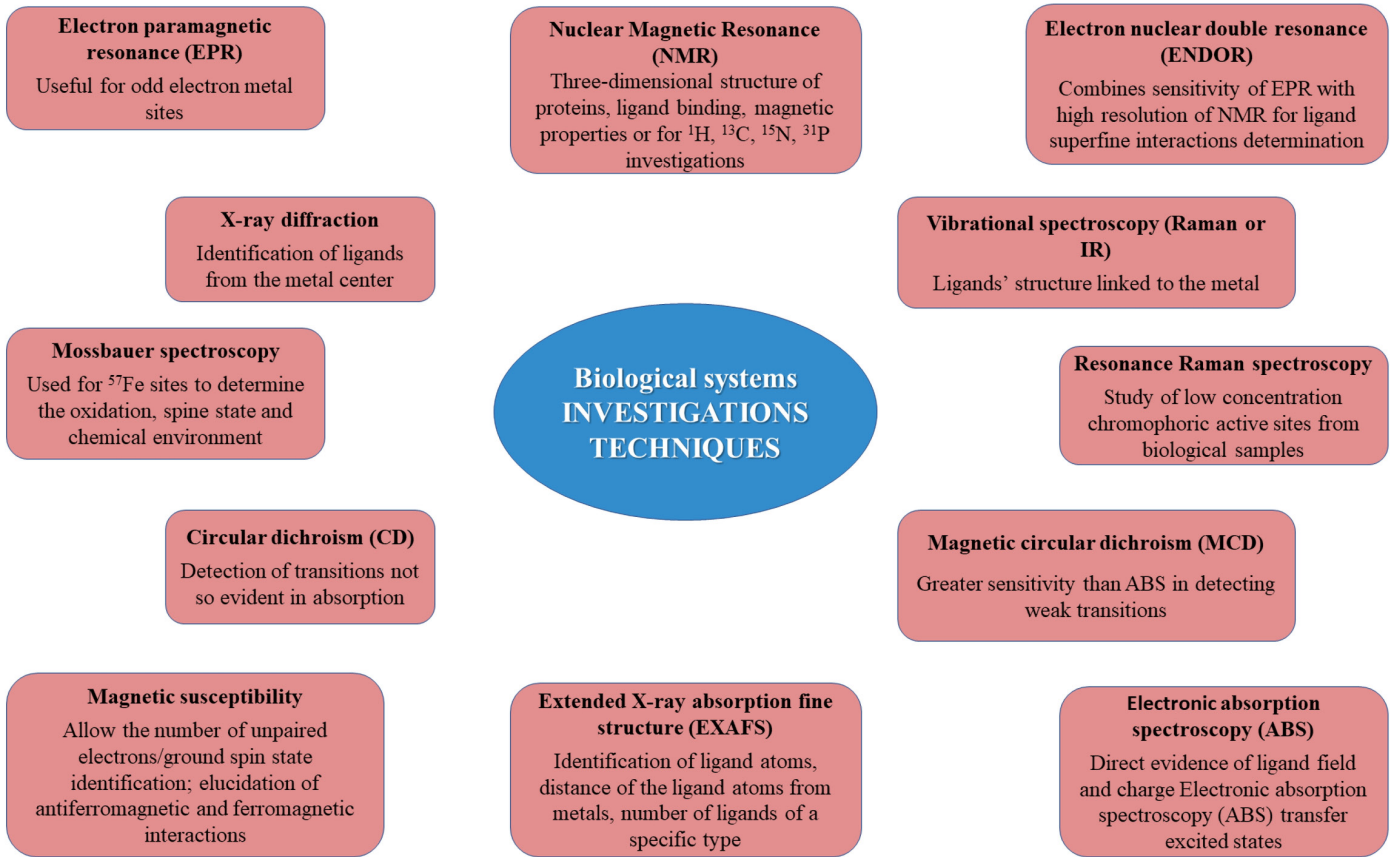
Methods for biological systems investigations and the appropriate information.

Several methods can be used for biological structures investigations. They allow the study of proteins or metals employed in the biological systems [5] (Figure 1).

In this work, we consider the further hypothesis as the key to explain the health and diseases relationship; it is presented in relation to today’s knowledge in the force fields, the appropriate evidence contained in the homeopathic Materia Medica [11], and our understanding regarding the levels of health. 

### The hypothesis

The hypothesis suggests that in every sudden stress (mental, emotional, or physical) that the organism is subjected to, there is a change in the overall structure of the energy field of the organism. Between the different electromagnetic changes due to pathology that are possibly occurring within the subatomic level, like ionized atoms, change in the number of electrons, change in the number of neutrons, quantum tunneling, quantum entanglement, electron excitons, the most probable and the most direct is a change in the spin of the electrons within one or more atoms on different elements. 

The spin of electrons [12], as long as the organism is in a well-balanced state, remains steady and stable and is difficult to change. But the spin changes not only under electromagnetic influences but also under strong stress like negative thoughts and emotions or under acute or chronic pathologies.

In the cases of a disease process, where symptoms start to appear, there are most probably changes in the spin of electron momentum from clockwise to anti-clockwise or the opposite, which indicates that the organism starts to be unbalanced. 

As long as this negative change of momentum persists, the organism remains in an unbalanced state. Therefore, a correct treatment should be one that will be able to bring back the spin to its original momentum when the organism was in a healthy state. 

The idea of electronic spin inversion has been considered as a danger for human health by Boswinkel [13], while in material science, the electron spin inversion has been proved in nanoscale graphene sheets [14] or gated silicene nanoribbons [15].

### Fields of force, the building blocks of life

On the other hand, today, we know with certainty that according to quantum physics, the force fields are also the building blocks of all living entities [16-18].

But what they call in homeopathy vital force is identifiable with the fields of forces that in quantum physics [19] are considered as the building blocks of all entities existing in the material world.

### The homeopathic remedy

The same is true concerning the homeopathic remedy. Its building blocks are the specific fields of force of the substance that we use to influence the organism in the level of vital force. After the process of potentization, through constant dilutions and succussions, the remedy arrives in a state of pure energy in the form of a specific field of force idiomorphic to the substance that is diluted and potentized [20].

Once the remedy reaches this energy state, then it can influence the subatomic particles that are connecting our material structure with the field of forces.

So, in homeopathic treatment, the remedy is acting on a subatomic level upon the so-called fields of force of the organism. It has to be understood that the homeopathic remedy can influence the vital force only by a force that is of a similar nature. We are actually creating such a force in the laboratory by the process of potentization of the remedy. 

In homeopathy, for instance, if an individual has sudden emotional stress like an unexpected rejection or a sudden and painful love disruption, a whole group of symptoms arises instantly, like extreme dryness of mouth, a sense of fainting, as if the blood is gone out through the feet as if everything is lost in life, a sense of deep isolation with strong palpitations, a sense of confusion and so on.

This kind of symptom group can be counteracted by a single homeopathic dose of Natrum-muriaticum in high potency. It seems that all the subatomic particles of the natrum element that are dispersed in the whole body, within the different organs, are instantly affected by an emotional or any other type of shock. Then, the correct remedy, selected from the homeopathic Materia Medica [11], will produce a kind of instant reset of the organism with all the relevant symptoms disappearing.

The obvious logical sequence then is to think that the potentized remedy, through its specific force field, has affected the vital collective force on account of its similarity [21] - the potentized remedy is formed in the proving upon the human organism, creating similar symptomatology, which is the basic law of homeopathy expressed as Similia Similibus Curentur.

It is this similarity of effect, an “entanglement” according to quantum physics [22], in the sub-atomic structure of NaCl, that the prescribing of Natrum-muriaticum in high potency would instantly reset the organism to normality. 

### The effect of pathological conditions upon the spin of electrons

Nevertheless, what part of the subatomic structure could be considered as most easily influenced by a strong negative effect through an emotional upset or a virus? 

The hypothesis suggests that the spin of the electrons will be the first to be influenced. In different other situations of stress, like a severe acute inflammatory condition or a financial shock or any stress from chemical drugs (the concept of organism-in-its-environment is known as the base of analysis when speaking about the living organisms [23]), that have a lasting effect, we also observe the instant manifestation of symptoms that cannot be accounted for, unless we perceive them as instant changes on an electromagnetic level within the human body.

In severe chronic pathologies, most probably, the spin of electrons of several atoms is affected simultaneously, creating deep distress within the organism; then, a condition of total chaos and loss of balance at a deep level is observed and after this, a structural pathology develops. 

It is an interesting experience for the homeopaths who apply the single individualized remedy, to confirm again and again [24-36], that if the organism is not totally confused, the indicated remedy appears to be clear, and its effect is instant and completely satisfying after a short initial therapeutic aggravation [37]. This condition - a clear remedy and an instant reset of the organism - indicates a good state of health, according to the theory of the Levels of Health [38].

On the contrary, in more complicated cases, where medical pathology has a long history, there was a deep initial trauma from a love disappointment, but its effect was not overcome by the organism, and the immune system was eventually weakened; then, a series of bronchitis that was not treated properly leads to subsequent development of a chronic asthmatic condition. We know that if the effect of the initial shock was left to grow for a long time without proper treatment, eventually, we observe the appearance of structural changes that lead to pathological changes recognized as chronic diseases.

Such a situation will obviously have a disturbance on several basic elements of the organism, for instance, (Na), calcium (Ca), zinc (Zn), magnesium (Mg), and sulfur (S). Some or all of them could be affected in their subatomic structure, resulting in a specific chronic disease. In these conditions, most probably, the spin of the electrons of all these elements will be forcefully influenced to change the direction of their spin. 

In such a case, electrons on different atoms become more and more affected, changing their spin, and the case becomes more and more complicated, difficult to recover, and the treatment must be very precise in the sequence of the remedies needed.

Precise prescribing implies that in such a case, there will be a need for several remedies over a long period and prescribed in a specific sequence [39].

Something which indicates that a cure, in such a deep pathology, involves prescribing a sequence of the indicated remedies that will be resetting step by step the spin of the electrons of the different elements involved.

Notably, a similar changing of the spin of electrons may occur from the electromagnetic waves of cell phones, which may cause deep disturbances if they are used extensively and close to the brain.

Likewise, a similar effect is suspected to happen for those who are living near or under high voltage electrical stations [40], where the electromagnetic waves may create such disturbances to the organisms [41] that could lead eventually to severe pathologies [42, 43], especially if the organisms are sensitive to such influences.

## Conclusions

Needless to say, if this hypothesis proves to be correct, the whole concept of conventional medicine, concerning diseases and their cure, will have to be changed drastically. 

The question, of course, remains: how could this change in the spin of electrons be detected and measured in a laboratory?

If there is such a confirmation, namely that a disease state is producing a change in the spin of electrons and the cure results only after we are able to affect the sub-atomic level of the vital force with a similar agent of an electromagnetic nature, we will have revolutionized medicine, far as the diagnosis and the cure of diseases is concerned.

We can then say that we have really entered the Epoch of Energy Medicine, where homeopathy belongs.

## Acknowledgment

The authors are grateful to professor Gulsen Onengut (Cukurova University, Adana, Turkey) for her valuable suggestions, discussion, and comments received during the preparation of this manuscript.

## References

[1] Atkins P., de Paula J (2006). Atkins’ Physical Chemistry.

[2] Griffiths D.J., Schroeter D.F. (2018). Introduction to quantum mechanics.

[3] Wertz J.E., Bolton J.R. (1986). Electron Spin Resonance. Elementary Theory and Practical Applications.

[4] Alberts B., Johnson A.D., Lewis J., Morgan D., Raff M., Roberts K. (2002). Molecular Biology of the Cell.

[5] Crichton R.R. (2012). Biological Inorganic Chemistry. A New Introduction to Molecular Structure and Function.

[6] Aslaksen E.W. (2008). Designing Complex Systems: Foundations of Design in the Functional Domain.

[7] Hacker C.F. (1948). Vital Force and Homœopathy. Br. Homeopath. J.

[8] McCraty R (2015). Science of the Heart. Exploring the Role of the Heart in Human Performance.

[9] Osborne T. (2016). Vitalism as Pathos. Biosemiotics.

[10] Waisse S., Bonamin L.V. (2016). Explanatory models for homeopathy: from the vital force to the current paradigm. Homeopathy.

[11] Kent J.T. (2002). Lectures on Homoeopathic Materia Medica.

[12] Guajardo G., Wilson J. (2005). Models for explaining the homeopathic healing process: a historical and critical account of principles central to homeopathy. Homeopathy.

[13] Boswinkel J. (2003). Electronic Spin Inversion: A Danger to Your Health. Explore.

[14] Ahmadi S., Esmaeilzadeh M., Namvar E., Pan G. (2012). Spin-inversion in nanoscale graphene sheets with a Rashba spin-orbit barrier. AIP Advances.

[15] Rzeszotarski B., Szafran B. (2018). Electron spin inversion in gated silicene nanoribbons.. Physical Review B.

[16] The Nobel Prize in Physics. (2013). The Royal Swedish Academy of Sciences.

[17] Grygar F. (2017). Bohr’s Complementarity Framework in Biosemiotics. Biosemiotics.

[18] Tong D. (2017). Quantum Fields: The Real Building Blocks of the Universe. https://www.youtube.com/watch?v=zNVQfWC_evg.

[19] Bernal G. (1993). Homœopathy and physics: A brief history. Br. Homeopath. J.

[20] Vithoulkas G. (1981). The science of homeopathy.

[21] Vithoulkas G. (1981). The science of homeopathy.

[22] Smith C.W. (2015). Electromagnetic and magnetic vector potential bio-information and water. Homeopathy.

[23] Cardenas-Garcia J.F., Ireland T. (2017). Human Distributed Cognition from an Organism-in-Its-Environment Perspective. Biosemiotics.

[24] Mahesh S., Mallappa M., Vithoulkas G. (2015). Gangrene: five case studies of gangrene, preventing amputation through homoeopathic therapy. Indian J. Res. Homoeopathy.

[25] Mahesh S., Mallappa M., Vithoulkas G. (2017). Embryonal carcinoma with immature teratoma: a homeopathic case report. Complemen. Med. Res.

[26] Mahesh S., Mallappa M., Tsintzas D., Vithoulkas G. (2017). Homeopathic treatment of vitiligo: a report of fourteen cases. Am. J. Case Rep.

[27] Tsintzas D., Vithoulkas G. (2017). Treatment of Postoperative Sore Throat with the Aid of the Homeopathic Remedy Arnica montana: A Report of Two Cases. J Evid. Based Complementary Altern. Med.

[28] Vacaras V., Vithoulkas G., Buzoianu A.D., Marginean. I., Major Z., Vacars V., Nicoara R.D., Oberbaum M. (2017). Homeopathic Treatment for Postpartum Depression: A Case Report. J. Evid. Based Complementary Altern. Med.

[29] Vithoulkas G., Vacaras V., Kavouras J., Buzoianu A.D., Marginean M., Vacaras D., Cozma S. (2017). Homeopathic treatment for prolonged postoperative coma: a case report. J. Med. Life.

[30] Chabanov D., Tsintzas D., Vithoulkas G. (2018). Levels of health theory with the example of a case of juvenile rheumatoid arthritis. J. Evid. Based Integr. Med.

[31] Denisova T.G., Gerasimova L.I., Pakhmutova N.L., Mahesh S., Vithoulkas G. (2018). Individualized Homeopathic Therapy in a Case of Obesity, Dysfunctional Uterine Bleeding, and Autonomic Dystonia. Am. J. Case Rep.

[32] Mahesh S., Mallappa M., Vithoulkas G. (2018). Could homeopathy become an alternative therapy in dengue fever? An example of 10 case studies. J. Med. Life.

[33] Tenzera L., Djindjic B., Mihajlovic-Elez O., Pulparampil B.J., Mahesh S., Vithoulkas G. (2018). Improvements in long standing cardiac pathologies by individualized homeopathic remedies: a case series. SAGE Open Med Case Rep.

[34] Mahesh S., Jaggi L., Jaggi A., Tsintzas D., Vithoulkas G. (2019). Individualised Homeopathic Therapy in ANCA Negative Rapidly Progressive Necrotising Crescentic Glomerulonephritis with Severe Renal Insufficiency – A Case Report. J. Med. Life.

[35] Mahesh S., Shah V., Mallappa M., Vithoulkas G. (2019). Psoriasis cases of same diagnosis but different phenotypes-management through individualized homeopathic therapy. Clin. Case Rep.

[36] Tsintzas T., Jaggi A., Jaggi L., Mahesh S., Vithoulkas G. (2019). Heterotopic ossification in a 7-year-old female patient treated with individualized homeopathy: A case report. Clin. Case Rep.

[37] Vithoulkas G. (1996). True but strange?. Nature.

[38] Vithoulkas G. (2019). Levels of Health.

[39] Vithoulkas G., Carlino S. (2010). The “continuum” of a unified theory of diseases. Med. Sci. Monit.

[40] Tourab W., Babouri A. (2016). Measurement and Modeling of Personal Exposure to the Electric and Magnetic Fields in the Vicinity of High Voltage Power Lines. Safety and Health at Work.

[41] Ohayon M.M., Stolc V., Freund F.T., Milesi C., Sullivan S.S. (2019). The potential for impact of man-made super low and extremely low frequency electromagnetic fields on sleep. Sleep Medicine Reviews.

[42] Doyon P.R., Johansson O. (2017). Electromagnetic fields may act via calcineurin inhibition to suppress immunity, thereby increasing risk for opportunistic infection: Conceivable mechanisms of action. Med. Hypotheses.

[43] Guo Y., Gu B.L., Zeng Z., Yu J.Z., Kawazoe Y. (2000). Electron-spin polarization in magnetically modulated quantum structures. Phys. Rev. B.

